# Monthly Record of Deaths in the City of Buffalo

**Published:** 1855-04

**Authors:** J. Root

**Affiliations:** Health Physician


### ART. XI.—*Report of Deaths in Buffalo for the month*

| DISEASES. | Males. | Females. | Total. | AGE. 1 year and under. Male. | Female. | 1 to 2 years. Male. | Female. |
|---|---|---|---|---|---|---|---|
| Albuminuria, | 1 | .... | 1 | .. | .. | .. | .. |
| Anasarca, | .... | 1 | 1 | .. | .. | .. | .. |
| Apoplexia, | .... | 1 | 1 | .. | .. | .. | .. |
| Abscess of Liver, | 1 | .... | 1 | .. | .. | .. | .. |
| Burned, | .... | 1 | 1 | .. | .. | .. | 1 |
| Consumption, | 11 | 13 | 24 | 1 | .. | .. | .. |
| Convulsions, | 11 | 10 | 21 | 6 | 6 | 2 | .. |
| Croup, | 5 | .... | 5 | 2 | .. | 1 | 1 |
| Child Bed, | .... | 1 | 1 | .. | .. | .. | .. |
| Congestion of Lungs, | .... | 1 | 1 | .. | .. | .. | .. |
| Cynanche Maligna, | .... | 1 | 1 | .. | .. | .. | 1 |
| Congestion of Brain, | 1 | .... | 1 | .. | .. | .. | .. |
| Chronic Peritonitis, | .... | 1 | 1 | .. | .. | .. | .. |
| Debility, | 2 | .... | 2 | 2 | .. | .. | .. |
| Dropsy, | 1 | 2 | 3 | .. | .. | .. | .. |
| " of the Brain, | .... | 3 | 3 | .. | .. | .. | 1 |
| " of the Heart, | .... | 1 | 1 | .. | .. | .. | .. |
| Dissipation, | 1 | .... | 1 | .. | .. | .. | .. |
| Diabetis, | 1 | .... | 1 | .. | .. | .. | .. |
| Diarrhœa, | 2 | .... | 2 | .. | .. | .. | .. |
| Dysentery, | .... | 1 | 1 | .. | .. | .. | .. |
| Exposure, | .... | 1 | 1 | .. | 1 | .. | .. |
| Encysted Tumor, | 1 | .... | 1 | .. | .. | 1 | .. |
| Fever Scarlet, | 2 | 3 | 5 | .. | 1 | 1 | 1 |
| " Typhus, | .... | 2 | 2 | .. | .. | .. | .. |
| " Typhoid, | 1 | .... | 1 | .. | .. | .. | .. |
| "Febris Nervosa," | 1 | .... | 1 | .. | .. | .. | .. |
| Frozen, | 1 | .... | 1 | .. | .. | .. | .. |
| Hydrocephalus, | 1 | 3 | 4 | 1 | .. | .. | 1 |
| Hydrothorax, | 1 | .... | 1 | .. | .. | .. | .. |
| Hepatitis, | .... | 1 | 1 | .. | .. | .. | .. |
| Hæmorrhage of Lungs, | .... | 1 | 1 | .. | .. | .. | .. |
| Hemiplegia, | .... | 1 | 1 | .. | .. | .. | .. |
| Hip Disease, | 1 | .... | 1 | .. | .. | .. | .. |
| Inflammation of Bowels, | 1 | .... | 1 | .. | .. | .. | .. |
| " " Brain, | 1 | 1 | 2 | .. | .. | 1 | .. |
| Marasmus, | 2 | .... | 2 | 1 | .. | .. | .. |
| Metro-peritonitis, | .... | 1 | 1 | .. | .. | .. | .. |
| Metritis, | .... | 1 | 1 | .. | .. | .. | .. |
| Old Age, | 4 | 1 | 5 | .. | .. | .. | .. |
| Organic disease of Brain, | 1 | .... | 1 | .. | .. | .. | .. |
| Premature, | 1 | .... | 1 | 1 | .. | .. | .. |
| Pneumonia, | 3 | 5 | 8 | 1 | 3 | .. | 2 |
| Scalded, | .... | 1 | 1 | .. | .. | .. | 1 |
| Small-pox, | 1 | .... | 1 | .. | .. | .. | .. |
| Suicide, | 2 | .... | 2 | .. | .. | .. | .. |
| Still-born, | 2 | 2 | 4 | .. | .. | .. | .. |
| Spine Disease, | 1 | .... | 1 | .. | .. | .. | .. |
| Typhoid Pneumonia, | 2 | .... | 2 | .. | .. | .. | .. |
| Unknown, | 8 | 5 | 13 | 7 | 3 | .. | 1 |
| Whooping Cough, | 3 | 3 | 6 | 2 | 2 | 1 | .. |
| Totals, | 78 | 69 | 147 | 24 | 16 | 7 | 10 |

NATIVITIES.—American, 45. German, 66. Irish, 19. French, 2. Swiss, 2

*of February,* 1855. By J. Root, M. D., Health Physician.

AGE.

| 2—5 | | 5—10 | | 10—15 | | 15—20 | | 20—25 | | 25—30 | | 30—40 | | 40—50 | | 50—60 | | 60—70 | | 70—80 | | 80—90 | | 90—100 | | 100 and upward. | | Not given. | |
|---|---|---|---|---|---|---|---|---|---|---|---|---|---|---|---|---|---|---|---|---|---|---|---|---|---|---|---|---|---|
| Male. | Female. | Male. | Female. | Male. | Female. | Male. | Female. | Male. | Female. | Male. | Female. | Male. | Female. | Male. | Female. | Male. | Female. | Male. | Female. | Male. | Female. | Male. | Female. | Male. | Female. | Male. | Female. | Male. | Female. |
| | | | | | | | | | | | | 1 | | | | | | | | | | | | | | | | | |
| | | | 1 | | | | | | | | | | | | | | | | | | | | | | | | | | |
| | | | | | | | | | 1 | | | | | | | | | | | | | | | | | | | | |
| | | | | 1 | | | | | | | | | | | | | | | | | | | | | | | | | |
| | | | | | | | | | | | | | | | | | | | | | | | | | | | | | |
| 1 | | | | | 1 | 2 | 2 | 1 | 2 | | 2 | 2 | 3 | 1 | 1 | 4 | 1 | | | | | | | | | | | | |
| 2 | 3 | 1 | 1 | | | | | | | | | | | | | | | | | | | | | | | | | | |
| | | 1 | | | | | | | | | | | | | | | | | | | | | | | | | | | |
| | | | | | | | | | | | | | 1 | | | | | | | | | | | | | | | | |
| | | | 1 | | | | | | | | | | | | | | | | | | | | | | | | | | |
| | | | | | | | | | | | | | | | | | | | | | | | | | | | | | |
| | | | | | | | | | | 1 | | | | | | | | | | | | | | | | | | | |
| | | | | | | | | | | | | | | | | 1 | | | | | | | | | | | | | |
| | | | | | | | | | | | | | | | | | | | | | | | | | | | | | |
| | | | | 1 | | | | | | | | | | | | | 1 | | | | 1 | | | | | | | | |
| | 1 | | | | | | | | | | | | | | 1 | | | | | | | | | | | | | | |
| | | | | | | | | | | | | | | | | | | | | | | | | | | | | 1 | |
| | | | | | | | | | | | | 1 | | | | | | | | | | | | | | | | | |
| | | | | | | | | | | | | | | | | | | 1 | | | | | | | | | | | |
| | | | | | | | | | | 1 | | | | 1 | | | | | | | | | | | | | | | |
| | | | 1 | | | | | | | | | | | | | | | | | | | | | | | | | | |
| | | | | | | | | | | | | | | | | | | | | | | | | | | | | | |
| | | | | | | | | | | | | | | | | | | | | | | | | | | | | | |
| 1 | 1 | | | | | | | | | | | | | | | | | | | | | | | | | | | | |
| | | | | | | | | | | | | | | | | | 1 | | | | 1 | | | | | | | | |
| | | | | | | | | | | | | | | 1 | | | | | | | | | | | | | | | |
| | | | | 1 | | | | | | | | | | | | | | | | | | | | | | | | | |
| | | | | | | | | | | | | | | | | | | 1 | | | | | | | | | | | |
| | | | | | 1 | | | | | | | | | | | | | | | | | | | | | | | | 1 |
| | | | | | | | | | | | | 1 | | | | | | | | | | | | | | | | | |
| | | | | | | | | | 1 | | | | | | | | | | | | | | | | | | | | |
| | | | | | | | | | | | | | | | | | | | 1 | | | | | | | | | | |
| | | | | | | | | | | | | | | | 1 | | | | | | | | | | | | | | |
| | | | | 1 | | | | | | | | | | | | | | | | | | | | | | | | | |
| | | | | | | | | | | | | | | | | 1 | | | | | | | | | | | | | |
| | | | | | | | | | | | | | | | | | | | | | | | | | | | | | 1 |
| | | | | | | | | | | | | | | | | 1 | | | | | | | | | | | | | |
| | | | | | | | | | 1 | | | | | | | | | | | | | | | | | | | | |
| | | | | | | | | | | | 1 | | | | | | | | | | | | | | | | | | |
| | | | | | | | | | | | | | | | | | | 3 | | 1 | | | | | 1 | | | | |
| | | | | | | | | | | | | 1 | | | | | | | | | | | | | | | | | |
| | | | | | | | | | | | | | | | | | | | | | | | | | | | | | |
| 1 | | | | | | | | | | | | | | | | | | | | | | | | | | | | 1 | |
| | | | | | | | | | | | | | | | | | | | | | | | | | | | | | |
| | | | | | | | | | | 1 | | | | | | | | | | | | | | | | | | | |
| | | | | | | | | | | | | 1 | | | | 1 | | | | | | | | | | | | | |
| | | | | | | | | | | | | | | | | | | | | | | | | | | | | | |
| | | | | 1 | | | | | | | | | | | | | | | | | | | | | | | | | |
| | | | | | | | | | | 1 | | 1 | | | | | | | | | | | | | | | | | |
| 1 | | | | | | | | | | | | | | | | | | | | | | | | | | | | | 1 |
| | | | | | | | | | | | | | | | | | | | | | | | | | | | | | 1 |
| 6 | 5 | 2 | 4 | 5 | 2 | 2 | 2 | 1 | 5 | 4 | 3 | 8 | 4 | 3 | 3 | 8 | 3 | 5 | 1 | 1 | 2 | 0 | 0 | 0 | 1 | 0 | 0 | 2 | 4 |

Colored, 1. Prussian, 1. Country not given, 11.—Total, 147.

